# Perioperative dynamics and significance of plasma-free amino acid profiles in colorectal cancer

**DOI:** 10.1186/s12893-018-0344-0

**Published:** 2018-02-21

**Authors:** Kayoko Katayama, Akio Higuchi, Hiroshi Yamamoto, Atsuko Ikeda, Shinya Kikuchi, Manabu Shiozawa

**Affiliations:** 1 Cancer Prevention and Control Division, Kanagawa Cancer Center Research Institute, 2-3-2, Nakao, Yokohama City, Asahi-ku 241-8515 Japan; 2 Department of Gastrointestinal Surgery, Kanagawa Cancer Center, Yokohama, Japan; 3 Research Institute for Bioscience Products & Fine Chemicals, Ajinomoto Co., Inc., Kanagawa, Japan

**Keywords:** Plasma-free amino acids, Colorectal cancer, AminoIndex Cancer Screening, Preoperative, Postoperative

## Abstract

**Background:**

For early detection of cancer, we have previously developed the AminoIndex Cancer Screening (AICS) system, which quantifies 6 plasma-free amino acids (PFAAs) in blood samples. Herein, we examined the usefulness of the AICS in patients with colorectal cancer (CRC) by comparing the preoperative and postoperative PFAA profiles.

**Methods:**

Our study cohort consisted of 62 patients who had undergone curative resection for CRC at our cancer center, with no recurrence at the time of the study. Blood samples were collected from fasted patients within 1 week before the resection and at 0.5–6.5 years post-resection. Following plasmapheresis, the PFAA levels were measured via liquid chromatography/mass spectrometry, and the AICS values were computed (the higher the value, the greater the probability of cancer). Risk was calculated from the AICS value and ranked as A, B, or C, with rank C representing the highest risk. All patients in our study were rank B + C.

**Results:**

The postoperative AICS value was lower than the preoperative value in 57 of the 62 patients; the rank was also lower postoperatively (49 patients, *p* < 0.001). The decline in both was stage-independent, even occurring in patients with right-sided tumors or poorly differentiated adenocarcinomas. For comparative purposes, the levels of 2 tumor markers (carbohydrate antigen 19–9 and carcinoembryonic antigen) were also examined; these were within the reference ranges in 70–80% of patients preoperatively and in 80–90% postoperatively.

**Conclusion:**

We suggest that tumor-bearing conditions alter the PFAA profiles, which may be used to predict prognosis and monitor for recurrence in CRC patients after tumor resection.

**Trial registration:**

This trial has been retrospectively registered at UMIN-CTR R000028005, Oct 06, 2016.

**Electronic supplementary material:**

The online version of this article (10.1186/s12893-018-0344-0) contains supplementary material, which is available to authorized users.

## Background

Colorectal cancer (CRC) is the third most commonly diagnosed cancer and the fourth leading cause of cancer-related death in the world. By 2030, its burden is expected to increase by 60% to more than 2.2 million new cases, with 1.1 million deaths [1, 2]. In Japan, CRC has a great societal impact [3].

The progression of CRC is generally slow, and its symptoms are not readily apparent, with almost no subjective symptoms in the early stages. Surgical resection is an effective treatment for CRC, and radical resection of localized tumors achieves high survival rates [4, 5]. However, the 5-year survival rate at stage IV is extremely poor (~ 18.8%) [6]. Moreover, CRCs recur after surgical resection in ~ 10% of patients with stage II disease and in ~ 20–40% with stage IIIa–IIIb disease; hence, recurrence remains an important concern [7, 8].

Recent advances in analytical techniques [e.g., liquid chromatography mass spectrometry LC/MS)] and metabolomics analysis (e.g., in vivo amino acid profiling) have been reported in the context of various diseases, including cancer. Plasma-free amino acid (PFAA) profiles have been shown to differ in healthy individuals and patients with cancer or other diseases, owing to complex metabolic changes [9, 10]. Based on our previously described “AminoIndex technology”, which assesses changes in PFAA levels via multivariate analysis [11, 12], we developed a cancer screening method termed “AminoIndex Cancer Screening (AICS)” [13, 14]. AICS is a novel means of evaluating the probability of cancer, and we are currently using it in clinical practice to screen for multiple cancer types.

AICS consists of a combination of 6 amino acids that are differentially expressed in different cancer types. It discriminates between changes in the levels of amino acids that are characteristic of a specific cancer type and those that are common among cancer types [13, 14], and statistically analyzes the differences. Based on the amino acid data, it evaluates the probability of the individual currently having cancer. Probability is ranked on a scale of 0.0–10.0, in which a higher number represents a higher probability. Risk is ranked as A, B, or C, with rank C representing the highest risk [14].

Although tumor markers such as carbohydrate antigen 19–9 (CA19–9) and carcinoembryonic antigen (CEA) can be easily quantified in blood tests, their levels may not be increased in early-stage cancers [15]. AICS detects cancer-related changes in amino acid profiles even in patients with stage I cancers, and the profiles do not change as the cancer progresses [14]. Moreover, because AICS is a simple examination requiring only blood sampling, it can be performed during regular health check-ups.

Although assessment of the clinical usefulness of AICS is ongoing, it remains unclear why the PFAA levels fluctuate (e.g., in response to tumor-bearing status) and whether such fluctuations trigger cancer. Further elucidation of the biological mechanisms underlying the changes in the PFAA levels might allow for the development of both static and dynamic models of carcinogenesis via system analysis. System analysis of cancer patients based on systemic amino acid metabolism provides information on the nature of the cancer and, by doing so, may not only facilitate early detection but also the formulation of treatment, prognostic, and relapse monitoring strategies.

In this preliminary study, the preoperative and postoperative PFAA profiles, which are indicative of tumor-bearing status, were compared in patients with CRC via AICS.

## Methods

### Study design

Our study cohort consisted of patients who underwent curative resection for primary stage 0–III CRC at the Department of Gastrointestinal Surgery, Kanagawa Cancer Center (KCC) between 2007 and 2014. Patients who already had undergone treatment for cancer, patients with a previous history of cancer, and patients with metastatic or recurrent cancer were excluded. Data from the amino acid research database of the KCC [13–15] were used for patients who had already completed PFAA concentration measurements during a previous study [13, 15, 16]. Only postoperative blood samples were collected for amino acid measurements in the present study.

Among the 124 patients who fulfilled the above criteria and had both preoperative and postoperative PFAA concentration measurements, 62 had a preoperative AICS rank of B + C; these 62 patients were the target of the preoperative and postoperative comparative analysis.

### Ethical considerations

This study was conducted in accordance with the Declaration of Helsinki, and the study protocol was approved by the KCC Research Ethical Committee. The attending physician provided each participant with an explanatory document and written consent form approved by the committee before the study. The content of the study was fully explained in writing and verbally, and voluntary written consent was obtained from all patients. The samples and data in this study were used for research and analysis after undergoing linkable anonymization.

### Blood collection and PFAA analysis

Preoperative blood samples were collected from hospitalized patients the morning before the operation. Postoperative blood samples were collected at least 6 months after the operation, based on the in vivo amino acid metabolism timeframe. All patients fasted for 8 h before blood sampling; postoperative blood samples were obtained in the outpatient blood collection room.

PFAA analysis was performed as described previously [13, 16]. Specifically, blood samples (5 mL) were drawn from the antecubital veins into tubes containing ethylenediaminetetraacetic acid disodium and immediately placed on ice. Plasma was separated from whole blood via centrifugation at 3000 rpm at 4 °C. The samples were then frozen and stored at − 80 °C until analysis. After the plasma collection, the PFAA profiles were determined at the Ajinomoto Institute for Innovation via liquid chromatography mass spectrometry [17–19].

The 6 amino acids used to detect CRC via AICS were serine, proline, valine, methionine, isoleucine, and lysine [13, 14] (Additional file 1). The probability of CRC was presented as the AICS value (0.0 to 10.0); AICS values of 5.0 and 8.0 had specificities of 80% and 95%, respectively. The higher the AICS value, the higher the CRC probability. For determining the risk of CRC based on the AICS value, 0.0–4.9 was classified as rank A, 5.0–7.9 as rank B, and 8.0–10.0 as rank C; the closer to rank C, the higher the CRC risk [14]. When referring to AICS in this article, we are specifically referring to AICS for CRC as opposed to AICS tests for other diseases, which examine different amino acid profiles.

### Measurement of the levels of tumor markers

Using serum samples from the study patients, the levels of the following 2 tumor markers were measured: CEA (chemiluminescence immunoassay, normal range ≤ 5.0 ng/ml) and CA19–9 (normal range ≤ 37.0 U/ml).

### The modified Glasgow prognostic score (mGPS)

We calculated the mGPS value using the C-reactive protein (CRP) and albumin (Alb) values from biochemical tests performed pre- and postoperatively. Subsequently, the mGPS was divided into 3 groups (I: Alb ≧3.5 g/dl and CRP ≦0.5 mg/dl, II: Alb < 3.5 g/dl or CRP > 0.5 mg/dl, III: Alb < 3.5 g/dl and CRP > 0.5 mg/dl) according to previous research [20].

### Statistical analyses

The χ^2^-test and t-test were used for comparative analyses of the patients’ demographics. The Wilcoxon signed-rank test was used to compare the preoperative and postoperative AICS values. GraphPad Prism 6 (GraphPad Software, Inc., San Diego, CA, USA) and R version 3.2.2 (The R Foundation for Statistical Computing, Vienna, Austria) software were used for all statistical analyses. A *p*-value of < 0.05 was considered statistically significant.

## Results

The demographics of the 62 patients in our study are shown in Table 1. Preoperatively, 25 patients were categorized as rank B and 37 as rank C. There were no significant differences in patient age or sex, tumor stage, location, differentiation status, or histological type, or treatment method between the preoperative rank B and C groups (Table 1). There was no tumor recurrence in our patient cohort.

**Table 1:** Patient demographics

		N (%) patients			p-value
		AICS rank B + C	AICS rank B	AICS rank C	
Number of cases		62	25	37	
Age, Years †	mean ± SD	63.1 ± 9.9	65.0 ± 9.5	61.8 ± 9.9	0.22
	(range)	(36–83)	(38–79)	(36–83)	
Sex ‡	male	35 (56.5)	13 (52.0)	22 (59.5)	0.75
	female	27 (43.5)	12 (48.0)	15 (40.5)	
TNM stage ‡	0	1 (1.6)	0	1 (2.7)	0.19
	I	17 (27.4)	9 (36.0)	8 (21.6)	
	II	21 (33.9)	5(20.0)	16 (43.2)	
	III	23 (37.1)	11(44.0)	12 (32.4)	
Tumor location ‡	right	17 (27.4)	7 (28.0)	10 (27.0)	0.07
	left	39 (62.9)	13 (52.0)	26 (70.3)	
	unknown	6 (9.7)	5 (20.0)	1 (2.7)	
Differentiation status ‡	well	15 (24.2)	5 (20.0)	10 (27.0)	0.57
	moderate	34 (54.8)	13 (52.0)	21 (56.8)	
	poor	3 (4.8)	1 (4.0)	2 (5.4)	
	other	10 (16.1)	6 (24.0)	4 (10.8)	
Histological type ‡	tub	43 (69.4)	15 (60.0)	28 (75.7)	0.10
	pap	5 (8.1)	2 (8.0)	3 (8.1)	
	pr	6 (9.7)	4 (16.0)	2 (5.4)	
	muc	3 (4.8)	0	3 (8.1)	
	other	5 (8.1)	4 (16.0)	1 (2.7)	
Treatment	resection	42 (67.7)	15 (60.0)	27 (73.0)	0.41
method ‡	resection + adj CT	20 (32.3)	10 (40.0)	10 (27.0)	

Abbreviations: AICS, AminoIndex Cancer Screening; SD, standard deviation; tub, tubular adenocarcinoma; pap, papillary adenocarcinoma; pr, poorly differentiated adenocarcinoma; muc, mucinous adenocarcinoma; adj CT, adjuvant chemotherapy

Comparison of rank B and C; † t-test; ‡ Fisher exact test

### Comparison of AICS values before and after resection of CRCs

**Fig. 1:**
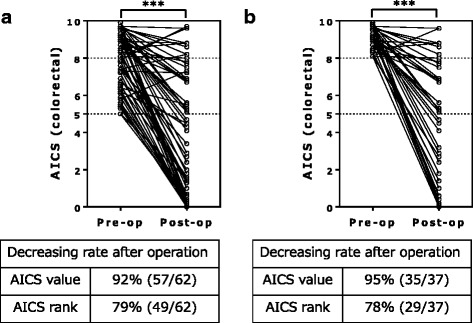
Differences between the preoperative and postoperative AminoIndex Cancer Screening (AICS) values according to rank. The paired preoperative and postoperative AICS values for ranks B + C (**a**) and C (**b**) are shown. The dotted lines show that the cut-off AICS values of 5 and 8 correspond to specificities of 80% and 95%, respectively. AICS: AminoIndex Cancer Screening. Pre-op: preoperative. Post-op: postoperative. ***p < 0.001, Wilcoxon signed rank test

Indicative of a lesser probability of cancer, 49 of the 62 patients in our study had a postoperative reduction in rank, and 57 had a postoperative reduction in the AICS value (*p* < 0.001) (Fig. 1a). In the analyses limited to preoperative rank C, the rank and AICS value declined postoperatively in 29 and 35 of 37 patients, respectively; the decrease in the AICS value was significant (*p* < 0.001) (Fig. 1b). Among all 62 patients, 5 showed a postoperative increase in the AICS value.

### Comparison of AICS values based on staging

The AICS values declined significantly after resection for disease stages I–III (p < 0.001; Table 2). A postoperative reduction was seen in either the AICS value, the rank, or both in patients at all stages, except in 5 stage II and III patients.

**Table 2:** Preoperative and postoperative AICS values and ranks according to clinicopathological features

	AICS value		p-value	Decrease in post-op AICS value, N (%)	Decrease in post-op AICS rank, N (%)
	Pre-op	Post-op			
Pre-op AICS rank B + C					
TNM stage					
0 (N = 1)	9.1	6.7	NA	1 (100)	1 (100)
I (N = 17)	7.7 ± 1.4	3.4 ± 2.4	< 0.001	17 (100)	15 (88)
II (*N* = 21)	8.2 ± 1.3	5.0 ± 3.5	< 0.001	18 (86)	15 (71)
III (*N* = 23)	8.0 ± 1.7	3.3 ± 3.6	< 0.001	21 (91)	18 (78)
Tumor location					
right (N = 17)	8.0 ± 1.6	2.3 ± 2.5	< 0.001	17 (100)	17 (100)
left (N = 39)	8.2 ± 1.4	4.7 ± 3.4	< 0.001	34 (87)	28 (72)
unknown (*N* = 6)	7.1 ± 1.5	2.2 ± 3.7	0.031	6 (100)	4 (67)
Differentiation status					
well (*N* = 15)	8.1 ± 1.5	4.3 ± 3.0	< 0.001	14 (93)	13 (87)
moderate (*N* = 34)	8.1 ± 1.5	3.8 ± 3.6	< 0.001	30 (88)	25 (74)
poor (*N* = 3)	8.0 ± 1.6	3.4 ± 2.9	0.25	3 (100)	3 (100)
other (*N* = 10)	7.8 ± 1.7	4.0 ± 2.9	0.002	10 (100)	8 (80)
Histological type					
tubular AC (*N* = 43)	8.1 ± 1.4	3.7 ± 3.3	< 0.001	39 (91)	36 (84)
papillary AC (*N* = 5)	7.6 ± 1.7	5.3 ± 3.0	0.063	5 (100)	3 (60)
poorly diff. AC (N = 6)	7.5 ± 1.2	4.3 ± 2.9	0.063	5 (83)	4 (67)
mucinous AC (N = 3)	9.6 ± 0.1	7.2 ± 1.8	0.25	3 (100)	2 (67)
other (N = 5)	7.1 ± 1.6	2.3 ± 4.1	0.063	5 (100)	4 (80)
Treatment method					
resection (*N* = 42)	8.1 ± 1.3	4.3 ± 3.1	< 0.001	38 (90)	33 (79)
resection + adj CT (*N* = 20)	8.0 ± 1.8	3.2 ± 3.6	< 0.001	19 (95)	16 (80)
Pre-op AICS rank C					
TNM stage					
0 (N = 1)	9.1	6.7	NA	1 (100)	1 (100)
I (*N* = 8)	9.0 ± 0.5	3.6 ± 2.5	0.008	8 (100)	8 (100)
II (*N* = 16)	8.8 ± 0.5	5.7 ± 3.1	0.001	14 (88)	11 (69)
III (*N* = 12)	9.5 ± 0.3	4.7 ± 3.6	< 0.001	12 (100)	9 (75)
Tumor location					
right (N = 10)	9.1 ± 0.5	3.0 ± 2.9	0.002	10 (100)	10 (100)
left (*N* = 26)	9.0 ± 0.6	5.6 ± 3.0	< 0.001	24 (92)	18 (69)
unknown (N = 1)	9.6	7.7	NA	1 (100)	1 (100)
Differentiation status					
well (N = 10)	9.0 ± 0.5	4.9 ± 2.9	0.002	10 (100)	9 (90)
moderate (N = 21)	9.0 ± 0.6	4.6 ± 3.6	< 0.001	19 (90)	15 (71)
poor (N = 2)	8.9 ± 0.3	5.1 ± 0.8	NA	2 (100)	2 (100)
other (N = 4)	9.6 ± 0.3	6.5 ± 2.1	0.125	4 (100)	3 (75)
Histological subtype					
tubular AC (*N* = 28)	9.0 ± 0.5	4.3 ± 3.2	< 0.001	26 (93)	24 (86)
papillary AC (N = 3)	8.7 ± 0.5	7.0 ± 2.5	0.25	3 (100)	1 (33)
poorly diff. AC (N = 2)	8.9 ± 0.3	5.1 ± 0.8	NA	2 (100)	2 (100)
mucinous AC (N = 3)	9.6 ± 0.1	7.2 ± 1.8	0.25	3 (100)	2 (67)
other (N = 1)	9.7	9.6	NA	1 (100)	0
Treatment method					
resection (*N* = 27)	8.9 ± 0.5	4.9 ± 3.1	< 0.001	25 (93)	22 (81)
resection + adj CT (N = 10)	9.6 ± 0.3	5.3 ± 3.6	0.002	10 (100)	7 (70)

AICS values are presented as the mean ± standard deviation. *p*-values are from the Wilcoxon signed-rank test. Abbreviations: AICS, AminoIndex Cancer Screening; Pre-op, preoperative; Post-op, postoperative; AC, adenocarcinoma; diff., differentiated; adj CT: adjuvant chemotherapy, NA: not applicable

### Comparison of AICS values based on tumor location

**Fig. 2:**
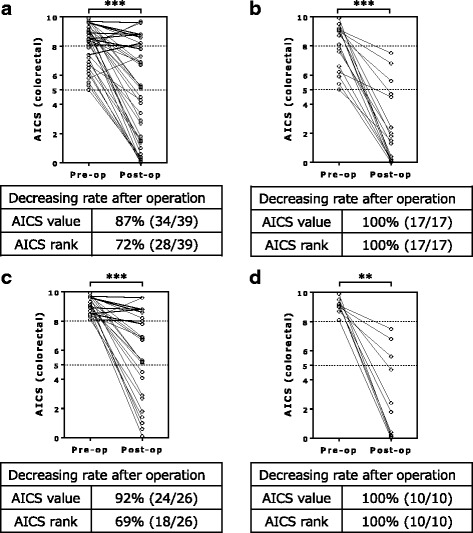
Differences between the preoperative and postoperative AICS values according to rank and tumor location. The paired preoperative and postoperative values for left- and right-sided tumors in the rank B + C group (**a** and **b**, respectively) and the rank C group (**c** and **d**, respectively) are shown. The dotted lines show that the cut-off AICS values of 5 and 8 correspond to specificities of 80% and 95%, respectively. AICS: AminoIndex Cancer Screening. Pre-op: preoperative. Post-op: postoperative. **p < 0.01, ***p < 0.001, Wilcoxon signed rank test

**Fig. 3:**
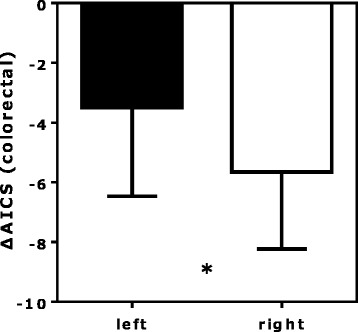
Differences between the preoperative and postoperative AICS values for left- and right-sided tumors. AICS, AminoIndex Cancer Screening. *p < 0.05, Student’s t-test

After resection, the AICS value and rank declined in all patients with right-sided tumors (*n* = 17) (Table 2). By contrast, the AICS value and rank did not decline in 11 and 5 patients with left-sided tumors (*n* = 39), respectively. The postoperative reduction in the AICS value was significantly greater in patients with right-sided tumors (preoperative value, 8.0 ± 1.6; postoperative value, 2.3 ± 2.5; *p* < 0.001) versus left-sided tumors (preoperative value, 8.2 ± 1.4; postoperative value, 4.7 ± 3.4; *p* < 0.001) (Table 2, Figs. 2 and 3).

### Comparison of AICS values based on histological type and degree of differentiation

After resection, the AICS values declined in some patients with tubular adenocarcinomas or moderately differentiated cancers (Table 2). However, the AICS values did not significantly decline in patients with other histological types or tumors with other degrees of differentiation.

### Tumor marker expression

Table 3 shows the preoperative and postoperative tumor marker levels. In the preoperative rank B + C group, the preoperative CEA level was below the cutoff value (≤5.0 ng/ml) in 43 (69.4%) patients and above the cutoff value (> 5.0 ng/ml) in 19 (30.6%) patients, whereas the postoperative CEA level was below and above the cutoff value in 55 (88.7%) and 7 (11.3%) patients, respectively (*p* = 0.015). The preoperative CA19–9 level was below the cutoff (≤37.0 U/ml) in 54 (87.1%) patients and above the cutoff value in 8 (12.9%) patients, whereas the postoperative CA19–9 level was above and below the cutoff value in 58 (93.5%) and 4 (6.5%) patients, respectively. Moreover, in the preoperative rank C group, the preoperative CEA and CA19–9 levels were below the cutoff value in approximately 90% of the patients; however, postoperatively, the differences were not significant. The accuracy of determination of these tumor markers was not high.

**Table 3:** Pre- and postoperative tumor marker (CEA and CA19–9) levels

	Pre-op AICS rank B + C (*N* = 62)			Pre-op AICS rank C (*N* = 37)		
	Pre-op	Post-op	p-value	Pre-op	Post-op	p-value
CEA, N (%)			0.015			0.021
> 5.0 ng/ml	19 (30.6)	7 (11.3)		12 (32.4)	3 (8.1)	
≤5.0 ng/ml	43 (69.4)	55 (88.7)		25 (67.6)	34 (91.9)	
CA19–9, N (%)			0.362			1.0
> 37.0 U/ml	8 (12.9)	4 (6.5)		2 (5.4)	1 (2.7)	
≤37.0 U/ml	54 (87.1)	58 (93.5)		35 (94.6)	36 (97.3)	

The p-value is for the comparison between pre-op and post-op values, Fisher’s exact test

Abbreviations: AICS, AminoIndex Cancer Screening; CEA, carcinoembryonic antigen; CA19–9, carbohydrate antigen 19–9; Pre-op: preoperative; Post-op: postoperative

### Changes in Alb, CRP, and the mGPS between the pre- and postoperative periods

Table 4 shows the preoperative and postoperative Alb (g/dl), CRP (mg/dl), and mGPS values. In the rank B + C group, the preoperative Alb level was approximately 4.0 g/dl, with no difference between the pre- and postoperative levels. The preoperative CRP level was high, at 0.4 ± 0.9 mg/dl, and decreased to 0.2 ± 0.2 mg/dl after surgery; however, a significant difference was not recognized. Regarding the mGPS, the percentage of patients classified as group I (Alb≧3.5 g/dl and CRP≦0.5 mg/dl) increased postoperatively, although no statistically significant difference was observed (Table 4).

**Table 4:** Changes in albumin, C-reactive protein, and the mGPS between the pre- and postoperative periods

	Pre-op AICS rank B + C (*N* = 24)			Pre-op AICS rank C (N = 15)		
	Pre-op	Post-op	p-value	Pre-op	Post-op	p-value
Alb (g/dl) †	4.0 ± 0.2	4.1 ± 0.4	0.278	4.0 ± 0.3	4.1 ± 0.3	0.174
CRP (mg/dl) †	0.4 ± 0.9	0.2 ± 0.2	0.220	0.6 ± 1.2	0.2 ± 0.2	0.130
mGPS, N (%)‡			0.701			0.330
I	19 (79.2)	21 (87.5)		11 (73.3)	14 (93.3)	
II	5 (20.8)	3 (12.5)		4 (26.7)	1 (6.7)	
III	0	0		0	0	

†Wilcoxon signed rank test; ‡ Fisher’s exact test

Abbreviations: AICS, AminoIndex Cancer Screening; Alb, albumin; CRP, C-reactive protein; mGPS, modified Glasgow Prognostic Score; Pre-op: preoperative; Post-op: postoperative. mGPS (3 groups): I: Alb ≧3.5 g/dl and CRP ≦0.5 mg/dl

II: Alb < 3.5 g/dl or CRP > 0.5 mg/dl

III: Alb < 3.5 g/dl and CRP > 0.5 mg/dl

### Relationship between the postoperative blood collection time point and the AICS value

There was no significant correlation between the time of postoperative blood collection and the AICS value in the 62 patients analyzed (r = − 0.083, *p* = 0.52; Additional file 2).

## Discussion

The amino acids in the plasma are maintained at constant levels by homeostatic processes in the body. Metabolomics analysis (e.g., in vivo amino acid profiling) of various disease states has shown alterations in the PFAA profiles owing to collapsed regulatory mechanisms; these disease states include cancer, liver failure, kidney failure, Alzheimer’s disease, and psychiatric disorders. Via metabolomics analysis, AICS statistically compares PFAA profiles between patients with CRC and healthy individuals; consequently, it can determine whether an individual has CRC. Although clinical application of this screening method has widened recently, the biological mechanisms that control the PFAA levels remain unknown, as does the cause and effect relationship between the PFAA levels and cancer. Hence, whether fluctuations in the PFAA levels cause cancer or vice versa has yet to be determined.

The present study showed a significant decline in the AICS values after surgical resection in patients with primary CRC and a preoperative rank of B + C. The decline was stage-independent, even occurring in patients with right-sided tumors or poorly differentiated adenocarcinomas, both of which are highly malignant (100% reduction in the AICS value and rank in both conditions). These results indicate that elimination of cancer cells restores the PFAA levels to precancer levels, as determined in vivo. Previous animal experiments have suggested that release of the nuclear protein HMGB1 into the blood, which affects the metabolism of distant organs, accounts in part for the altered PFAA levels in cancer patients [21]. Alterations may also result from interactions between cancer cells, with involvement of the immune system [22]. However, assessment of postoperative changes using the mGPS, a prognostic indicator, showed no significant difference in this study, although the proportion of patients classified as group I tended to increase.

The results of this study suggest that changes in the PFAA levels in patients with CRC strongly reflect CRC-bearing conditions; i.e., the cancer causes the changes in the PFAA levels. This point should be further clarified, as recurrence of CRC after resection is an important problem.

Tumor markers are often used to monitor for relapse after CRC resection. However, in the present study, the sensitivities of CEA and CA19–9 for detecting CRC were low, similar to in a previous study of early-stage CRC [15]. Moreover, these markers were significantly less sensitive than AICS for the detection of CRC [15].

Although previous studies have compared the preoperative and postoperative PFAA profiles in patients with breast, stomach, and thyroid cancers [23, 24], there have been no comparable investigations in patients with CRC. We previously observed changes in the levels of 18 different amino acids after treatment of various cancers; however, the interpretation of the results was cumbersome, because some amino acids increased in abundance whereas others decreased [23]. In the present study, the use of only 6 amino acids (identified via the AminoIndex technology using multivariate analysis scores) for determination of AICS values simplified interpretation of the data and provided information regarding the probability of cancer.

Recent reports have indicated that right-sided CRCs are more malignant than left-sided CRCs and have a worse prognosis [25]. The complexity of the colorectal region and the different characteristics of left-sided versus right-sided CRCs have stimulated discussions about the selection of therapeutic agents [26]. In 2016, the American Society of Clinical Oncology reported that many of the variables associated with right-sided tumors were major indicators of poor prognosis [27, 28]. According to previous reports, 20–30% of CRCs occur on the right side and 70–80% on the left side [6]. Similarly, in our study, about 60% of CRCs with a rank of B or C were on the left side [29]. The results of our study show that resection of highly malignant right-sided tumors significantly reduces the AICS value. This finding suggests that assessment of treatment efficacy is necessary at earlier stages for right-sided CRCs compared with left-sided CRCs.

Our study examined changes in the preoperative and postoperative PFAA profiles in patients with CRC via AICS, which is a cancer probability assessment test. We suggest that cancer cells alter the PFAA profiles, which is reflected in the AICS value. This premise has important clinical implications but requires verification.

Several aspects of our study require discussion and further testing. First, in 5 patients, the AICS value did not decline after resection. All 5 patients were classified with left-sided CRC, and postoperative adjuvant chemotherapy was administered to 4 of these cases. The four cases of moderately differentiated cancers demonstrated a deeper invasion depth than the one case with highly differentiated cancer; many cases have been previously reported showing vascular invasion, lymph node metastasis, and peritoneal dissemination [30]. In regard to the one case of highly differentiated type, it is necessary to collect and verify similar cases in the future. Although combining surgery and chemotherapy is effective for these high-risk cases, the efficacy of postoperative adjuvant chemotherapy for stage II CRC has not yet been established in Japan. Moreover, implementation of adjuvant chemotherapy largely depends on hospital policy and thus currently differs among hospitals [4]. Two of the above 5 patients were confirmed to have recurrence postoperative (Additional file 3). However, for the remaining 3 cases, it remains unknown why the AICS value did not decrease.

Second, the present study excluded subjects classified as AICS rank A. These patients showed no postoperative changes in their PFAA profiles, which were within the AICS tolerance range for sensitivity, specificity, and positive predictive value (Additional file 4). However, investigations that consider factors such as patient background, history of other diseases, and drug compliance status are needed.

Third, the timing of postoperative follow-up blood collection should be considered. Because this study was an exploratory study, blood was collected 6 or more months after resection, but the range varied widely, from 0.5–6.5 years (median, 4.1 years). However, there was no significant correlation between the AICS value and the blood collection time (Additional file 2). Furthermore, the relationship between amino acid metabolism, the PFAA profile, and the amount of time between surgical trauma and wound healing is unknown [22]. In this study, only 5 cases showed recurrence during the follow-up. In the future, the number of cases will be increased prospective verification with predetermined blood collection time points and long-term studies with follow-up until recurrence are needed.

This study showed that the PFAA profile reflects the tumor-bearing status in patients with CRC. AICS might be an effective way to predict prognosis and monitor recurrence and the patient’s clinical course postoperatively.

In the future, it will be essential to clarify through ongoing research whether elimination of factors indicative of poor prognosis affects the degree of AICS value reductions.

## Conclusions

AICS is a simple means of monitoring CRC risk, requiring only a small volume of blood. Moreover, the results of the present study suggest that it might also be used to predict prognosis and monitor for recurrence in CRC patients after tumor resection. Hence, AICS might reduce the incidence of postoperative recurrence by facilitating earlier detection of CRCs.

## Additional files

Additional file 1: **Figure S1.** Changes in amino acids contained in the AICS formula before and after colorectal cancer resection (comparison with healthy people). Axis: Area under the ROC curve discriminating between healthy people and patients with CRC for each amino acid. Abbreviations: Pre-op, preoperative; Post-op, postoperative; AICS, AminoIndex Cancer Screening. (PPTX 108 kb)

Additional file 2: **Figure S2.** Correlation between the period of postoperative blood collection and the AICS value. Spearman rank correlation coefficient: r = − 0.083 (− 0.333~ 0.178), *p* = 0.52. Abbreviations: AICS, AminoIndex Cancer Screening. (PPTX 91 kb)

Additional file 3: **Figure S3.** Recurrent cases after postoperative AICS (colorectal) measurement. a: Pre-op rank B + C, b: Pre-op rank C. Wilcoxon signed rank test: *p* > 0.05. Abbreviations: Pre-op, preoperative; Post-op, postoperative; AICS, AminoIndex Cancer Screening; n.s., not significant. (PPTX 102 kb)

Additional file 4: **TableS1.** Characteristics of rank A patients. (DOCX 21 kb)

## Abbreviations

AICS: AminoIndex Cancer Screening
Alb: Albumin
CA19–9: Carbohydrate antigen 19–9
CEA: Carcinoembryonic antigen
CRC: Colorectal cancer
CRP: C-reactive protein
KCC: Kanagawa Cancer Center
mGPS: Modified Glasgow Prognostic Score
PFAA: Plasma-free amino acid
