# Supplementary figures and images for: Perioperative dynamics and significance of plasma-free amino acid profiles in colorectal cancer
Source: BMC Surg. 2018 Feb 21;18:11. doi: 10.1186/s12893-018-0344-0 (PMC5822659; doi:10.1186/s12893-018-0344-0)

## Slide 1
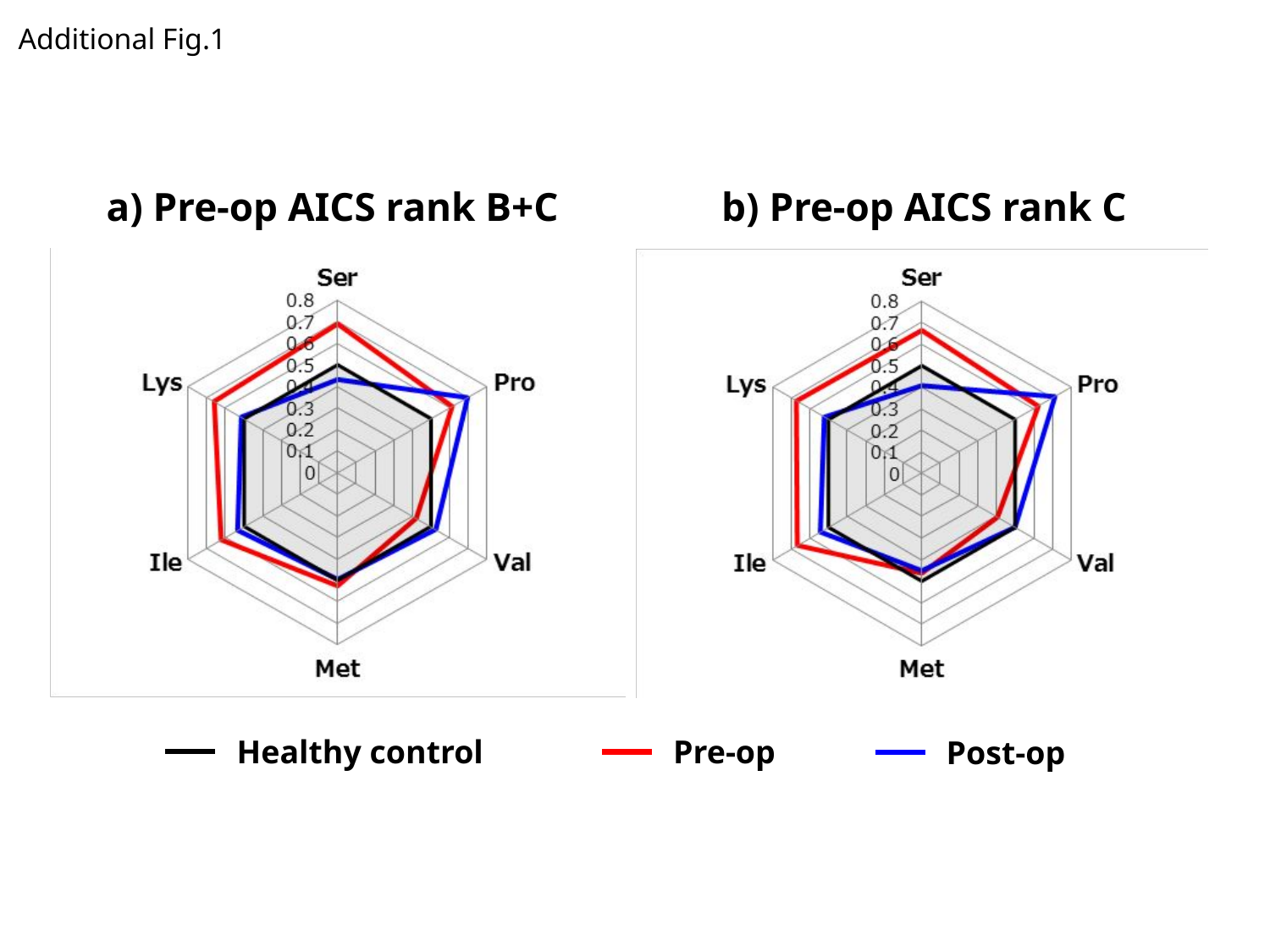

Additional Fig.1
a) Pre-op AICS rank B+C
b) Pre-op AICS rank C
Healthy control
Pre-op
Post-op

Supplement: Supplementary file 1 — Figure S1. Changes in amino acids contained in the AICS formula before and after colorectal cancer resection (comparison with healthy people). Axis: Area under the ROC curve discriminating between healthy people and patients with CRC for each amino acid. Abbreviations: Pre-op, preoperative; Post-op, postoperative; AICS, AminoIndex Cancer Screening. (PPTX 108 kb) [file 12893_2018_344_MOESM1_ESM.pptx]

## Slide 1
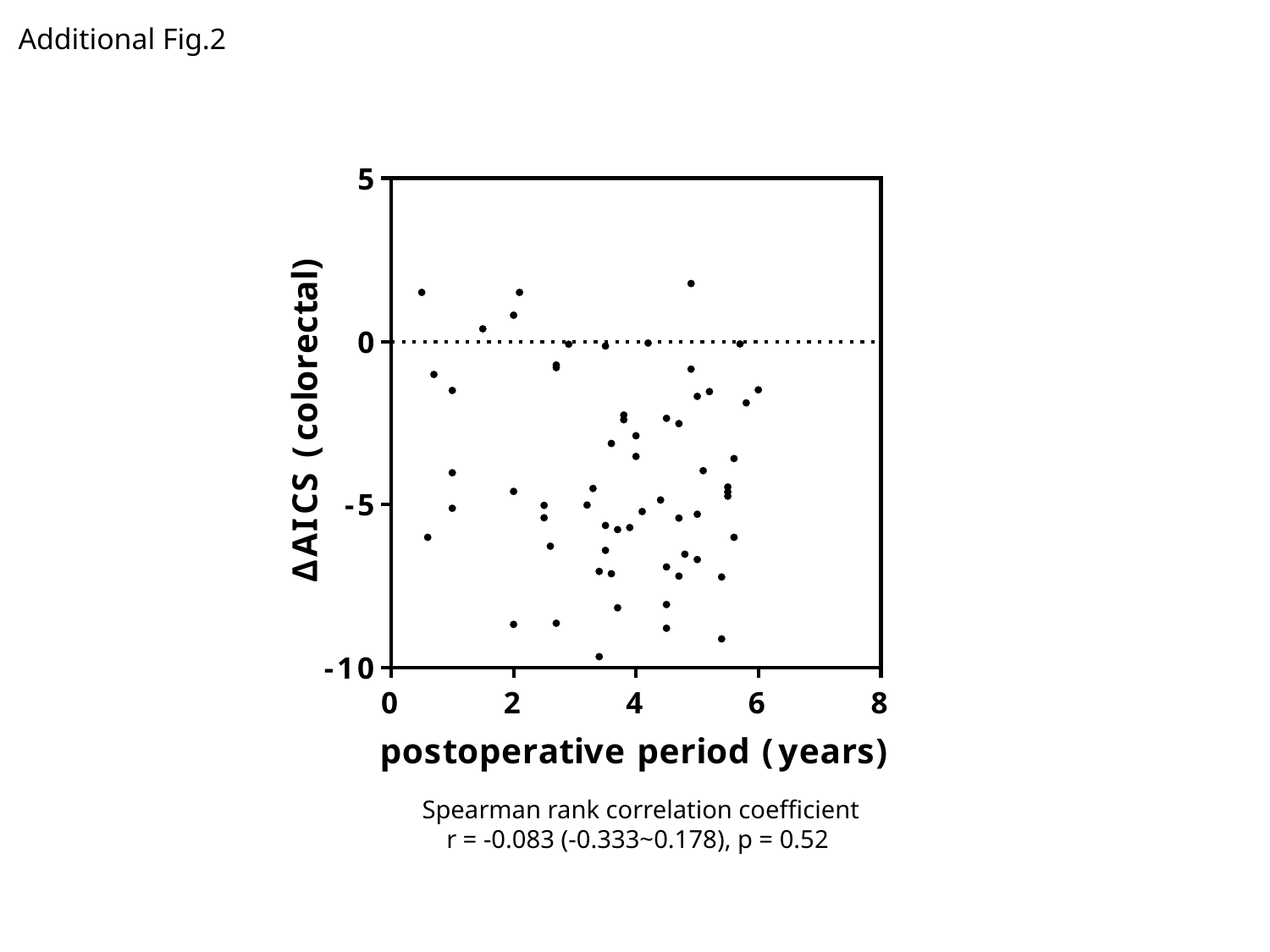

Additional Fig.2
Spearman rank correlation coefficient
r = -0.083 (-0.333~0.178), p = 0.52

Supplement: Supplementary file 2 — Figure S2. Correlation between the period of postoperative blood collection and the AICS value. Spearman rank correlation coefficient: r = − 0.083 (− 0.333~ 0.178), p = 0.52. Abbreviations: AICS, AminoIndex Cancer Screening. (PPTX 91 kb) [file 12893_2018_344_MOESM2_ESM.pptx]
