# Supplementary material for: Perioperative dynamics and significance of plasma-free amino acid profiles in colorectal cancer
Source: BMC Surg. 2018 Feb 21;18:11. doi: 10.1186/s12893-018-0344-0 (PMC5822659; doi:10.1186/s12893-018-0344-0)
Supplement: Supplementary file 3 — Figure S3. Recurrent cases after postoperative AICS (colorectal) measurement. a: Pre-op rank B + C, b: Pre-op rank C. Wilcoxon signed rank test: p > 0.05. Abbreviations: Pre-op, preoperative; Post-op, postoperative; AICS, AminoIndex Cancer Screening; n.s., not significant. (PPTX 102 kb) [file 12893_2018_344_MOESM3_ESM.pptx]

## Slide 1
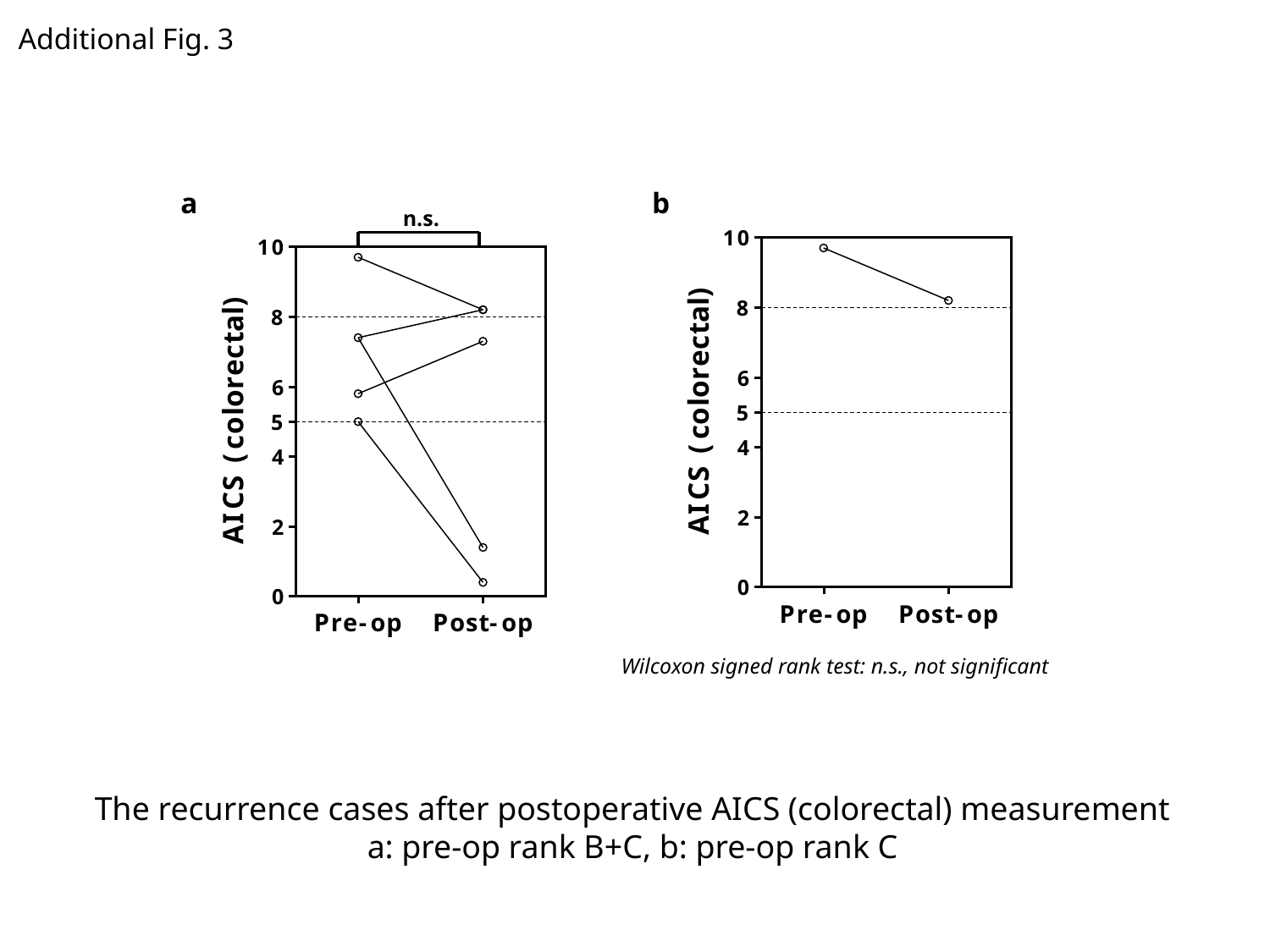

Additional Fig. 3
b
a
n.s.
Wilcoxon signed rank test: n.s., not significant
The recurrence cases after postoperative AICS (colorectal) measurement
a: pre-op rank B+C, b: pre-op rank C
