# Supplementary material for: Perioperative dynamics and significance of plasma-free amino acid profiles in colorectal cancer
Source: BMC Surg. 2018 Feb 21;18:11. doi: 10.1186/s12893-018-0344-0 (PMC5822659; doi:10.1186/s12893-018-0344-0)
Supplement: Supplementary file 4 — TableS1. Characteristics of rank A patients. (DOCX 21 kb) [file 12893_2018_344_MOESM4_ESM.docx]

| NO | pre AIC value | pre AICS lank | CEA | CA19-9 | Stage | Primary tumor | Degree of differentiation | Histological type | Operation date | post-CEA | post-CA19-9 |
| --- | --- | --- | --- | --- | --- | --- | --- | --- | --- | --- | --- |
| 1 | 3.2 | A | 1.6 | 6 | 3 | A | Unknown | Tubular adenocarcinoma | 2007/3/19 | <0.5 | 4.9 |
| 2 | 4.6 | A | 2.2 | 4.6 | 3(S)、0(R) | S,R | well-differentiated(S)、unknown(R) | Tubular adenocarcinoma(S)、Adenomatous polyps in the carcinoma in situ ( R ) | 2007/4/9 | 3.3 | 4.9 |
| 3 | 2.3 | A | 1.3 | 7.8 | 3 | RS | well-differentiated | Papillary adenocarcinoma | 2007/4/20 | 1.2 | 10.5 |
| 4 | 2.2 | A | 2.2 | 3.8 | 3 | - | moderately differentiated | Tubular adenocarcinoma | 2007/5/25 | 2.6 | 4.2 |
| 5 | 3.7 | A | 1 | 4.4 | 2 | A | well-differentiated | Papillary adenocarcinoma | 2007/5/28 | 1.2 | 6.1 |
| 6 | 3.7 | A | 1.4 | 7.9 | 2 | R | well-differentiated | Tubular adenocarcinoma | 2007/6/25 | 1.8 | 11.0 |
| 7 | 3 | A | 1.8 | 4.2 | 1 | C | Unknown | Tubular adenocarcinoma | 2007/7/5 | 3.1 | 4.6 |
| 8 | 1.4 | A | 2.5 | 32.8 | 3 | S | moderately differentiated | Tubular adenocarcinoma | 2007/7/6 | 2.1 | 32.7 |
| 9 | 3.1 | A | 2.8 | 11 | 1 | RS | well-differentiated | Tubular adenocarcinoma | 2007/8/17 | 2.9 | 11.3 |
| 10 | 3.1 | A | 1.3 | 7.3 | 3 | S | moderately differentiated | Tubular adenocarcinoma | 2007/8/30 | 0.9 | 8.8 |
| 11 | 1.2 | A | 2.8 | 16.3 | 3 | R | well-differentiated | Tubular adenocarcinoma | 2007/9/28 | 3.3 | 16.7 |
| 12 | 0.6 | A | 1.9 | 12.9 | 2 | R | well-differentiated | Tubular adenocarcinoma | 2007/10/19 | 1.8 | 17.1 |
| 13 | 1.5 | A | 1.2 | 10.3 | 1 | C | moderately differentiated | Tubular adenocarcinoma | 2007/10/29 | 1.5 | 13.6 |
| 14 | 2.7 | A | 1.2 | 1.5 | 3 | C | moderately differentiated | Tubular adenocarcinoma | 2007/10/31 | 1.8 | 2.8 |
| 15 | 1.7 | A | 2.8 | 8 | 2 | S | moderately differentiated | Tubular adenocarcinoma | 2007/11/5 | 3.7 | 8.2 |
| 16 | 2 | A | 3.7 | 63.5 | 3 | R | well-differentiated | Tubular adenocarcinoma | 2007/11/9 | 1.5 | 44.4 |
| 17 | 0.4 | A | 1.4 | 6.4 | 2 | S | poorly differentiated | Poorly　ferentiated adenocarcinoma | 2007/11/28 | 0.8 | 5.8 |
| 18 | 4 | A | 2 | 29.5 | 1 | R | moderately differentiated | Tubular adenocarcinoma | 2007/11/26 | 2.2 | 123.0 |
| 19 | 0.4 | A | 1.3 | 5.9 | 1 | R | well-differentiated | Tubular adenocarcinoma | 2007/11/30 | 1.3 | 5.9 |
| 20 | 3.8 | A | 2.4 | 4.5 | 3 | A | moderately differentiated | Tubular adenocarcinoma | 2007/12/10 | 1.6 | 6.1 |
| 21 | 1.9 | A | 1.1 | 10.2 | 1 | S | moderately differentiated | Tubular adenocarcinoma | 2008/2/1 | 1.1 | 10.7 |
| 22 | 4.4 | A | 2.9 | 52 | 3 | A | Unknown | Tubular adenocarcinoma | 2008/1/31 | 3.2 | 53.0 |
| 23 | 3 | A | 1.2 | 10 | 3(D)、1(S) | D,S | well-differentiated(D, S) | Tubular adenocarcinoma (D, S) | 2008/2/28 | 1.2 | 15.7 |
| 24 | 1.1 | A | 22.2 | 7.8 | 2 | S | moderately differentiated | Tubular adenocarcinoma | 2008/3/21 | 3.6 | 7.9 |
| 25 | 3.4 | A | 1.7 | 4.7 | 3 | S | moderately differentiated | Tubular adenocarcinoma | 2008/6/9 | 2.4 | 5.1 |
| 26 | 4.5 | A | 1.8 | 8.2 | 2 | A | moderately differentiated | Tubular adenocarcinoma | 2008/7/4 | 2.1 | 11.0 |
| 27 | 0.9 | A | 11.8 | 1 | 3 | R | moderately differentiated | Adenocarcinoma | 2008/8/4 | 1.4 | <1.0 |
| 28 | 3.1 | A | 1.2 | 3 | 1 | S | moderately differentiated | Tubular adenocarcinoma | 2008/9/12 | 1.2 | 7.0 |
| 29 | 2.2 | A | 2.5 | 44.8 | 3 | C | Unknown | Tubular adenocarcinoma | 2008/10/17 | 4.2 | 42.0 |
| 30 | 4.7 | A | 1.3 | 3.9 | 1 | R | moderately differentiated | Tubular adenocarcinoma | 2008/10/24 | 1.6 | 4.2 |
| 31 | 4.5 | A | 2.5 | 8.9 | 4 | S | moderately differentiated | Tubular adenocarcinoma | 2008/12/22 | 2.1 | 8.6 |
| 32 | 0.1 | A | 2 | 29 | 3 | A | poorly differentiated | Poorly differentiated adenocarcinoma | 2008/12/24 | 2 | 17.3 |
| 33 | 1.4 | A | 1.2 | 1 | 1 | RS | well-differentiated | Tubular adenocarcinoma | 2009/1/22 | 0.8 | <1.0 |
| 34 | 2.6 | A | 49.6 | 7.1 | 2 | T | moderately differentiated | mucinous cancer | 2009/1/23 | 3.4 | 5.9 |
| 35 | 0.1 | A | 1.7 | 7.1 | 3 | RS | moderately differentiated | Tubular adenocarcinoma | 2009/1/30 | 0.9 | 13.8 |
| 36 | 4.4 | A | 4.2 | 31.2 | 1 | A | well-differentiated | Tubular adenocarcinoma | 2009/2/6 | 3.4 | 27.8 |
| 37 | 4.9 | A | 1.3 | 6.9 | 2 | S | well-differentiated | Tubular adenocarcinoma | 2009/2/16 | 1.4 | 6.9 |
| 38 | 0.4 | A | 1.3 | 8 | 1 | RS | Unknown | Unknouwn | 2009/3/2 | 1.2 | 10.0 |
| 39 | 4.7 | A | 9.2 | 35.1 | 3 | R | moderately differentiated | Tubular adenocarcinoma | 2009/3/30 | 5 | 27.5 |
| 40 | 0.1 | A | 1.1 | 5 | 1 | S | well-differentiated | Tubular adenocarcinoma | 2009/4/20 | 1 | 6.2 |
| 41 | 2.9 | A | 3.9 | 12.1 | 3 | R | moderately differentiated | Tubular adenocarcinoma | 2009/5/22 | 1.8 | 13.6 |
| 42 | 0.4 | A | 2.3 | 1 | 2 | - | moderately differentiated | Tubular adenocarcinoma | 2009/6/1 | 2.5 | <1.0 |
| 43 | 0.1 | A | 1.1 | 3 | 2 | S | moderately differentiated | Tubular adenocarcinoma | 2009/6/4 | 0.5 | 3.2 |
| 44 | 1.7 | A | 3.1 | 10.3 | 2 | RS | moderately differentiated | Tubular adenocarcinoma | 2009/6/8 | 1.1 | 5.9 |
| 45 | 2.8 | A | 1.4 | 4.5 | 1 | S | moderately differentiated | Tubular adenocarcinoma | 2009/6/22 | 2.3 | 5.4 |
| 46 | 1.3 | A | 1.1 | 7.7 | 2 | RS | well-differentiated | Papillary adenocarcinoma | 2009/6/26 | 1 | 8.8 |
| 47 | 3.1 | A | 5.6 | 15 | 1 | C | well-differentiated | Tubular adenocarcinoma | 2009/9/11 | 4.8 | 22.9 |
| 48 | 2.8 | A | 0.6 | 7.9 | 1 | R | moderately differentiated | Tubular adenocarcinoma | 2009/10/26 | 0.8 | 8.0 |
| 49 | 1.9 | A | 0.5 | 8.8 | 1 | - | well-differentiated | Tubular adenocarcinoma | 2009/11/13 | 0.5 | 5.2 |
| 50 | 4.5 | A | 5.4 | 4.6 | 3 | D | well-differentiated | Tubular adenocarcinoma | 2009/11/19 | 1.3 | 7.8 |
| 51 | 0.3 | A | 0.9 | 8.5 | 1 | A | moderately differentiated | Tubular adenocarcinoma | 2009/12/11 | 1.4 | 9.6 |
| 52 | 4.1 | A | 6.3 | 17.9 | 2 | D | moderately differentiated | Tubular adenocarcinoma | 2009/12/18 | 8.6 | 20.4 |
| 53 | 2.5 | A | 24 | 20.5 | 3 | RS | Unknown | - | 2009/12/18 | 2.9 | 17.6 |
| 54 | 0.2 | A | 2.9 | 7.2 | 3 | A | moderately differentiated | Tubular adenocarcinoma | 2009/2/22 | 2.2 | 6.2 |
| 55 | 2.9 | A | 2.8 | 4.5 | 2 | S | moderately differentiated | Tubular adenocarcinoma | 2009/3/25 | 2.9 | 5.3 |
| 56 | 3 | A | 0.7 | 34.2 | 3 | R | poorly differentiated | Poorly differentiated adenocarcinoma | 2009/4/5 | 0.8 | 6.9 |
| 57 | 3.3 | A | 3.2 | 18.6 | 1 | D | Unknown | Papillary adenocarcinoma | 2009/4/16 | 3.2 | 23.6 |
| 58 | 3.8 | A | 4.1 | 10 | 2 | T | moderately differentiated | Tubular adenocarcinoma | 2009/5/20 | 4.7 | 11.6 |
| 59 | 3 | A | 1.7 | 19.6 | 3 | R | Unknown | Poorly differentiated adenocarcinoma | 2009/6/18 | 0.9 | 10.0 |
| 60 | 3.1 | A | 3.3 | 8.1 | 1 | T | well-differentiated | Tubular adenocarcinoma | 2009/6/21 | 5.9 | 8.9 |
| 61 | 0.5 | A | 2.3 | 4.9 | 2 | A | well-differentiated | Tubular adenocarcinoma | 2009/7/26 | 2.7 | 4.6 |
| 62 | 4.8 | A | 2.7 | 22 | 1 | C | moderately differentiated | Tubular adenocarcinoma | 2009/9/2 | 2.9 | 27.7 |

**Abbreviations:** AICS, AminoIndex Cancer Screening; CA19-9, carbohydrate antien 19-9; CEA, carcinoembryonic antigen.

A, Ascending colon; T, Transverse colon, D, Descending colon; S, Sigmoid colon; RS, Rectosigmoid junction; R, Rectum.
